# Coexistence of territorial competitor ants in fragmented boreal forest landscape

**DOI:** 10.1007/s00442-024-05626-8

**Published:** 2024-09-28

**Authors:** Jouni Sorvari, Esa Huhta, Harri Hakkarainen

**Affiliations:** 1 Natural Resources Institute Finland, Latokartanonkaari 9, 00790 Helsinki, Finland; 2 Department of Biology, University of Turku, 20014 Turku, Finland; 3 Natural Resources Institute Finland, Ounasjoentie 6, 96200 Rovaniemi, Finland

**Keywords:** Boreal forests, Forest species, Competition, Competitive exclusion, *Formica aquilonia*, *Formica polyctena*, Forest fragmentation, Temporary habitat loss

## Abstract

The distribution of species in a patchy habitat may be influenced by competitive interactions. The dominant and highly competitive boreal ant species belong to the *Formica rufa* group. A pair of species, *Formica aquilonia* and *Formica polyctena*, require extensive territories due to their multi-nest breeding habits. The coexistence and habitat patterns of these two wood ant species in the boreal forest landscape were investigated. Forest characteristics in the vicinity of nests in forest patches were similar for both species, but they did not coexist in the same sampling plots of 0.79 ha in forest patches, indicating competitive exclusion. The sampling plots in large forest patches were more occupied by *F. aquilonia*, while no such association was found for *F. polyctena*. At a larger spatial scale (78.5 ha), we found that *F. polyctena* was more tolerant of smaller forest patches than *F. aquilonia* suggesting that these two ant species can coexist in moderately fragmented forest landscapes. However, forest habitat loss, fragmentation and climate-induced changes in forest tree structure may shift the species balance in favour of *F. polyctena* over *F. aquilonia* in the future.

## Introduction

Competition between species is widespread in nature and attracts extensive research in ecology based on experimental and theoretical approaches. Landscape structure can affect the survival of populations through interspecific interactions, particularly competition (Hanski 1995). In this context, dominant competitors may exclude each other from the same habitat patch. Consequently, interspecific competition may increase the risk of extinction of subpopulations (Hanski 1998).

The coexistence of species that use the same niche can be attained in a fragmented ecosystem when the competitively superior species has limited dispersal capability (Tilman 1994; Tilman et al. 1994) or when the inferior species exhibits a lower intrinsic rate of extinction than the superior species (Nee and May 1992; Moilanen and Hanski 1995). Several mathematical models have been published concerning the regional coexistence of competitors (Nee and May 1992; Dytham 1995; Moilanen and Hanski 1995; review by Moquet et al. 2001; Tilman et al. 1997; Doncaster et al. 2003), although data on coexistence of competitors at the habitat landscape scale are limited. To our knowledge, very few such studies have been conducted (e.g., Hanski and Ranta 1983).

Habitat loss is a principal cause of species extinction globally (Ehrlich and Ehrlich 1981; Simberloff 1984; Wilson 1988). Expansion of urban settlements and agricultural areas decrease forest coverage and increase the distance between forest patches. Forest management have similar effects of forest coverage and fragmentation, yet in shorter temporal scale. This fragmentation can result in populations being divided into several sub-populations that are connected via dispersing sexuals. Due to the habitat loss and fragmentation, several abundant species may become rare and go extinct. Models indicate that when habitat loss occurs, it may result in time-delayed species extinction, referred to as extinction debt, where the natural demographic functions of the population are no longer sustainable (Tilman et al. 1994; Hanski 2000; Hanski and Ovaskainen 2002). Tilman et al. (1994) suggest that the gradual and predictable disappearance of organisms is most likely in the case of dominant and efficient resource users, which can lead to increased intra- and inter-specific resource competition (Samia and Lutscher 2010; Cheng et al. 2019).

Boreal ants have a clear three-level hierarchy of competition namely, territorials, aggressive non-territorials, and submissives (Vepsäläinen and Pisarski 1982; Savolainen and Vepsäläinen 1988; Savolainen et al. 1989). Specifically, submissive species defend their nests only, whilst aggressive non-territorials defend their nests as well as their sources of food. On the other hand, territorials defend a larger territory around their nests and food sources, thereby guarding against intruding rivals (Savolainen et al. 1989). Mound-building red wood ants of the *Formica rufa* group demonstrate behavioural dominance within the boreal and temperate forests of Europe, commonly found inhabiting coniferous and mixed forest stands (Risch et al. 2016; Vandegehuchte et al. 2017; Sondej et al. 2021). This group of ants is classified as territorials within a three-level dominance hierarchy scheme, indicating their probable competition for habitats. In Finland, two of the red wood ant species, *F. aquilonia* Yarrow, 1955 and *F. polyctena* Förster, 1850, build multi-nest colonies and therefore require larger forest patches compared to single-nest breeding species. Therefore, these ants offer a sound testing ground to examine the impacts of competition on both the habitat patch level and the wider landscape level.

*Formica aquilonia* and *F. polyctena* are at the top of the interspecific competition hierarchy, suggesting competition for habitat and inability to tolerate each other in the vicinity of their colonies (Savolainen et al. 1989). In a forest stand in Eastern Finland, Kilpeläinen et al. (2008) found evidence of spatial separation between *F. aquilonia* and *F. polyctena* at forest patch level. Additionally, comparable spatial separation was noted in an isolated southwest Finnish forest patch in Loimaa region (J. Sorvari, unpublished). In the latter case, there existed a 100-m space devoid of mounds among multi-nest colonies of the species, while in Eastern Finland, the zone was narrower but still distinguished by the absence of mounds. In a third case, wood ants were surveyed on Lake Konnevesi islands (Sorvari 2018) where *F. aquilonia* and *F. polyctena* never co-occurred on a same island. Based on their territoriality and spatially wide multi-nest colonies these species furnish opportunities for studying the effects of interspecific competition at the landscape level. This paper presents data collected over a large area on two competing ant species coexisting in a fragmented boreal forest landscape. The aim of this study is to investigate whether the two ant species coexist within the same forest patch, whether they have different nesting habitat characteristics and habitat associations, and whether their probability of occurrence differs in relation to the amount of forest habitat.

## Materials and methods

### Study area

We investigated the presence of red wood ants in 133 forest patches within a 1150 km^2^ area surrounding the rural region of Laukaa and Konnevesi Research Station in Central Finland (62°37′N, 26°20′E) during the summers of 1999–2000. The study area is situated within boreal coniferous forest naturally fragmented by mires, lakes, and rivers that has been further fragmented by human induced clear-cuts, agricultural fields, and roads. The forests comprise mainly of Norway spruce (*Picea abies*), interspersed with varying proportions of Scots pine (*Pinus sylvestris*) and birches (*Betula* sp.).

### Study species

The study species, *Formica aquilonia* and *F. polyctena*, belong to the wood-ant group (*Formica rufa* group) with five species occurring in Finland. Their colonies are mainly polygynous and polydomous, with multiple egg-layers in a nest. Multiple nests collaborate to share and defend a large foraging territory, which usually includes interiors and margins of large coniferous forest patches (Collingwood 1979; Rosengren and Pamilo 1983; Mabelis 1994; Punttila 1996; Sorvari 2022). The three other species in the *F. rufa* group present in Finland, *F. rufa* Linnaeus, 1761, *F. lugubris* Zetterstedt, 1838, and *F. pratensis* Rezius, 1783 typically establish single-nest colonies (monodomy) in open forests, young forests, small forest fragments, or at the edges of forests (Rosengren and Pamilo1983; Punttila et al. 1994; Punttila 1996; Sorvari 2022).

*Formica aquilonia* is a species that is typically found in the northern and high-altitude regions of Europe. In contrast, its competitor *F. polyctena* has a more southern distribution (Collingwood 1979; Douwes et al. 2012; Seifert 2018). While there is a significant overlap in the distribution ranges of these two species in Finland, the presence of *F. polyctena* is clearly less common at high latitudes (Sorvari 2021, 2022). The primary food sources for both species under examination are comparable, comprised of aphid honeydew excretions and arthropod prey (Domisch et al. 2016). They also build large dome-shaped nest mounds of various organic matter, including conifer needles, small branches, sand and soil particles, and resins (Lenoir et al. 1999; Frouz et al. 2016).

### Forest landscape structure

In this study, the term ‘forest patch’ refers to relatively discrete patches with timber volume over 50 m^3^ ha^−1^ that can be separated from younger successional stages or other types of habitats (mires, lakes agricultural fields) comprising the landscape matrix around forest patches. The landscape structure was initially determined visually as being more, intermediate, or less fragmented based on aerial photographs. Less fragmented landscapes consisted of large forest patches that were more or less connected to each other. Intermediately fragmented landscapes had higher connectivity between forest patches than had more fragmented landscapes. The aim was to create a comprehensive continuum of different landscape types, from continuous to fragmented. We used 3 × 3 km sampling frame as a tool to provide a reliable assessment of landscape structure. Landscape structure was determined using aerial photographs where the sampling frame was centred on a forest patch. This central forest patch was used in the study and will hereafter be referred to as the ‘focal patch’. This sampling frame method allowed us to include in the study a wider analysis of landscape structure within a 500 m radius (78.5 ha). The selection of a focal patch with the sampling frame was repeated a total of 133 times to include an appropriate number of different landscape types. The minimum size of the local patch was approximately 5 ha. An area with a radius of 500 m (78.5 ha) from the focal patch was employed for analyses of forest landscape structure and represents landscape level in this study.

To acquire variables for landscape structure, we utilised classified Landsat TM5 images from the years 1995 to 1997 that were created by the National Land Survey of Finland (NLS; Vuorela 1997). Forest and land-use data, based on satellite images, were imported into a Geographic Information System (GIS). The landscape structure variables obtained were total forest cover % (percentage of landscape covered by forest with timber volume ≥50 m^3^ ha^−1^), spruce forest cover %, pine forest cover %, deciduous tree cover %, mixed forest cover %, and cover % of advanced thinning forest % (here after ATF). The cut point for ATF irrespective of forest type was based on the total volume of timber over 102 m^3^ ha^−1^, which is somewhat lower than the average volume and age reported for advanced thinning stage in Central Finland by Tomppo et al. (1999).

The 500 m radius circle typically consisted of several forest patches, and non-forested land types. Forest patches within this radius were defined from satellite image pixels (25 × 25 m) that have timber volume over 50 m^3^ ha^−1^. Using this criterion, a single forest patch was clearly separable from the neighbouring open habitats and sapling areas in the field. The focal patch size and the mean nearest-neighbor distance over all forest patches of the same successional type were utilized as landscape variables and calculated via the FRAGSTATS program (McGarigal and Marks 1995).

An index for forest habitat amount was formed from three intercorrelated habitat metrics through a principal component analysis (Table 1). Only the first principal component (PC) was utilised for the three variables: ATF cover (%), forest cover (%), and focal forest patch size (ha), resulting in a gradient from a fragmented forested habitat at negative scores to a more continuous forested habitat at positive scores (Table 1). The resulting index is referred to as the forest habitat amount gradient.

**Table 1:** Principal component loadings (PC1) for the habitat amount index at the landscape level radius of 500 m

Index composition	PC loading
*Habitat amount index* (79.79% of variance)	
% of forest cover	0.94
% ATF cover	0.83
Focal patch size (ha)	0.91

The focal patch refers to the patches with ant sampling plots and ATF refers to at least advanced thinning forest stage with the total timber volume over 102 m^3^ ha^−1^. Forest cover refers to coverage of forest with timber volume ≥50 m^3^ ha^−1^

It is worth noting that while the foraging distance of a large red wood ant nest is reported to exceed 100 m (Sorvari 2009), effective foraging distance is generally considered closer to 30 m (Niemelä and Laine 1986). It can be reasonably assumed that this 500 m radius area will encompass the entire territory of a polydomous colony and its surrounding foraging area. This makes it a suitable measure for landscape-level effects on the focal patch.

### Ant sampling plots

The ant sampling plots were 0.79 ha in size, with a 50 m radius, i.e., 100 m in diameter, and were situated in the middle of each focal patch. The ant sampling plots were defined as being occupied or empty. If occupied, we recorded the species, number of mounds and the basal area of nests (see Table 2). Basal area of nest mound can be used as a proxy for worker population size of the mound (e.g., Seifert 2016). We sometimes found nests outside the ant sampling plots and in most of the cases occurred along occupied ant sampling plots. For species identification, we collected around 20 workers from each nest and utilized the keys provided by Collingwood (1979) and Douwes et al. (2012).

**Table 2:** Number of occupied ant sampling plots out of the 133 ant sampling plots surveyed and total number of nests

	Occupied plots (*N*)	Total *N* of nests	Cumulative nest basal area (m^2^)	Mean nest basal area (m^2^)	Mean nest numbers (*N*)
*F. aquilonia*	61/133	146	3.76 ± 2.98	1.31 ± 0.86	2.86 ± 1.78
*F. polyctena*	17/133	20	2.65 ± 2.13	1.77 ± 1.04	1.54 ± 0.63

Corresponding means (±SD) for cumulative basal area of nest mounds, mean basal area of nests, and number of nest mounds within occupied ant sampling plots for *F. aquilonia* and *F. polyctena* in the study area

We found 146 nests of *F. aquilonia*, 20 nests of *F. polyctena*, 8 nests of *F. lugubris*, 4 nests of *F. rufa*, and no nests of *F. pratensis*. After combining all 133 occupied and unoccupied ant sampling plots, totalling 104.4 ha, the nest densities (ha^−1^) of wood ant species were: *F. aquilonia* 1.40, *F. polyctena* 0.19, *F. lugubris* 0.04, *F. rufa* 0.02, and *F. pratensis* 0.00. These densities differ from the values measured by Punttila and Kilpeläinen (2009) most probably due to the smaller geographical coverage in our study. The monodomous species *F. lugubris* and *F. rufa* were found at the edge of forest within ant sampling plots. One third of the monodomous nests (two nests of *F. lugubris*) occurred within ant sampling plot occupied by *F. aquilonia* and *F. polyctena* (one in each species). Therefore, we concluded that their impact on the competition between *F. aquilonia* and *F. polyctena* at the landscape level was negligible.

### Forest characteristics of ant sampling plots

Forest stand characteristics of ant sampling plots were measured in the field within four non-overlapping circles, each having a radius of 4 m (50 m^2^). The circles were placed in four cardinal directions 30 m from the centre of each ant sampling plot. Measurements from the four circles were averaged to an ant sampling plot level (radius of 50 m). The variables of forest characteristics at an ant sampling plot area included mean density of tree trunks (ha^−1^), mean diameter of tree trunks at breast height (cm), mean stem volume (m^3^ ha^−1^), mean stem basal area (m^2^ ha^−1^) and mean age of trees. We recorded the total numbers of trees by species that were larger or equal to 9.5 cm diameter at breast height. We estimated the tree ages by using local fresh cut tree stumps in nearby clearcut areas of all tree species encountered in ant sampling plots. First the circumference of tree stump and its accurate age from tree rings were regressed. We then interpolated the age of each tree from the regression slopes (see Suorsa et al. 2003). The tree species consisted of Norway spruce (*Picea abies*), Scots pine (*Pinus sylvestris*), birches (*Betula pubescens*, *B. pendula*), and other deciduous trees (e.g., *Salix caprea*, *Populus tremula*).

### Statistical analyses

The analyses were conducted on two nested spatial scales; at the ant sampling plot level (radius of 50 m) and at the landscape matrix level (radius of 500 m). Statistical analyses were conducted using SAS 9.4 software (SAS Institute Inc., Cary, NC, USA). The likelihood-ratio chi-square test (PROC FREQ) was utilized to analyse the possibility of competitive exclusion. In addition to this, differences in forest habitat variables between these two ant species were analysed by using the discriminant analysis (PROC DISCRIM). Proportions of different tree species, stem diameter (cm), stem density, timber volume and forest age were analysed.

Differences in the amount of forest habitat between *F. aquilonia*, *F. polyctena* patches and unoccupied patches were assessed using a generalised linear model (PROC GLIMMIX) with normal distribution and the identity link function. Variances were corrected using RANDOM_RESIDUAL_/GROUP = occupancy type. Single-predictor landscape variable models and probabilities for species occupation (0 = not occupied, 1 = occupied) were analysed using PROC GLIMMIX with normal distribution and the identity link function for forest fragmentation and forest habitat loss gradients.

## Results

### Coexistence of species

**Fig. 1:**
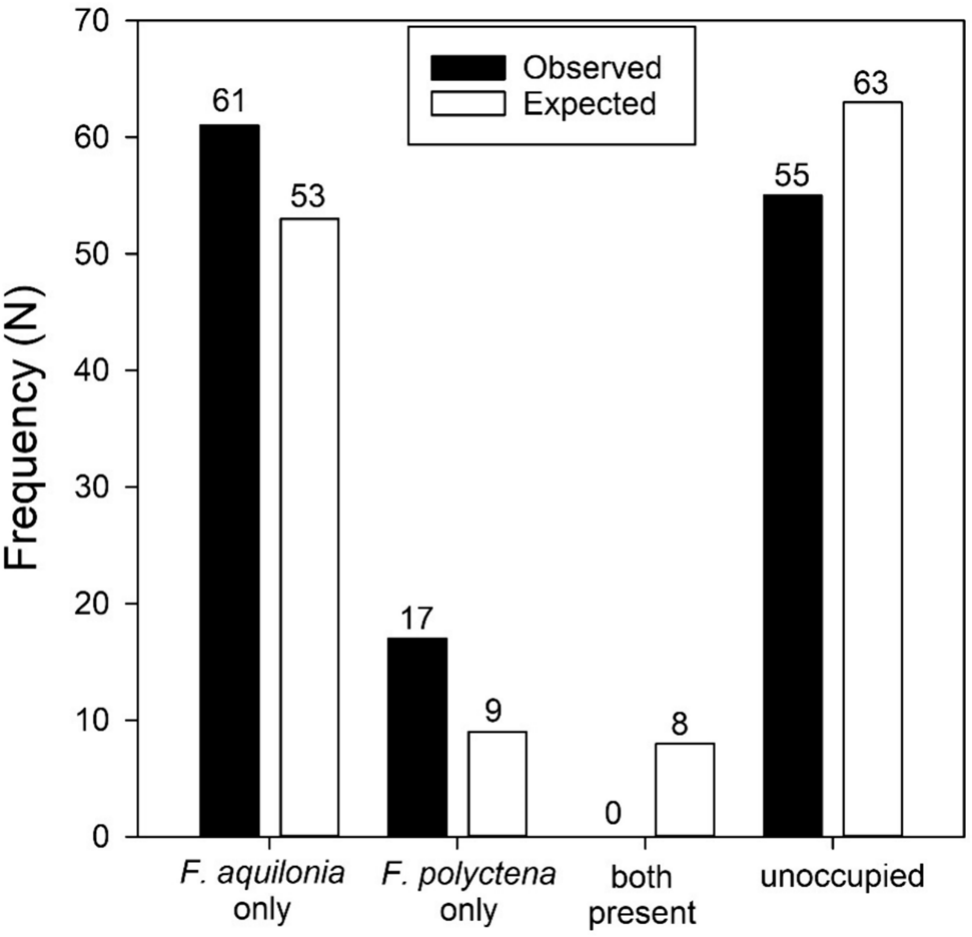
The observed frequency of *F. aquilonia*, and *F. polyctena*, and the combined frequency of both species in ant sampling plots with a radius of 50 alongside the frequencies expected by the model. It is noteworthy that the species did not coexist in the same sampling plot

*Formica aquilonia* occupied 61 of the 133 ant sampling plots in the study area, while *F. polyctena* was found in 17 ant sampling plots. In addition, the total number of observed nests was higher in *F. aquilonia* than in *F. polyctena* (146 vs. 20). The species were never found occurring together in the same ant sampling plots. Neither of the species was present in 55 sampling plots. We conducted a likelihood ratio chi-square test to assess the independence of the species’ distribution patterns. The distributions of ant species were dependent on each other, and they did not occur together in the same sampling plots (*N* = 133, df = 3, *G*^2^ = 14.70, *p* = 0.0021; Fig. 1).

To test whether the species differ in forest characteristics, a discriminant analysis was conducted (*F. aquilonia* 61 patches, *F. polyctena* 17 patches, *N* = 78). There were no significant differences in the factors between the species (Table 3), and their ability to discriminate was also not significant (Wilk’s *λ*: 0.96, *F*_8, 69_ = 0.33, *p* = 0.96). It is evident that characteristics of the forest habitats of *F. aquilonia* and *F. polyctena* did not differ, and they did not coexist in the same ant sampling plots, indicating a competitive exclusion between the two species. Ant sampling plots occupied by either species differed significantly from unoccupied plots only by the percentage of other deciduous trees (Table 4). The ability of the set of variables to discriminate between occupied and unoccupied sampling plots was not significant (Wilk’s *λ*: 0.92, *F*_9, 122_ = 1.17, *p* = 0.32).

**Table 3:** Percentage of trunks by tree species, trunk diameters, trunk densities, stem volumes, stem basal areas, and estimated mean tree age (mean ± 95% confidence intervals) in ant sampling plots 50 m of radius occupied by *Formica aquilonia* (*N* = 61) and *F. polyctena* (*N* = 17) colonies

	Mean ± CI		Univariate test
	*F. aquilonia*	*F. polyctena*	
*Picea abies* %	74.6 ± 4.2	72.9 ± 10.2	*F*_1, 76_ = 0.13, *p* = 0.72
*Pinus sylvestris* %	12.2 ± 3.5	13.3 ± 7.7	*F*_1, 76_ = 0.09, *p* = 0.77
*Betula* sp. %	10.6 ± 2.7	9.8 ± 5.8	*F*_1, 76_ = 0.08, *p* = 0.78
Other deciduous trees %	2.6 ± 1.4	4.0 ± 3.4	*F*_1, 76_ = 0.79, *p* = 0.38
Mean trunk diameter (cm)	25.2 ± 0.9	25.5 ± 2.1	*F*_1, 76_ = 0.11, *p* = 0.74
Mean trunk density (ha^−1^)	984.4 ± 84.8	935.3 ± 201.1	*F*_1, 76_ = 0.27, *p* = 0.60
Mean stem volume (m^3^ ha^−1^)	322.7 ± 28.0	324.3 ± 57.6	*F*_1, 76_ < 0.01, *p* = 0.96
Mean stem basal area (m^2^ ha^−1^)	46.9 ± 2.8	45.7 ± 7.5	*F*_1, 76_ = 0.14, *p* = 0.71
Mean tree age (years)	49.1 ± 2.3	50.0 ± 5.2	*F*_1, 76_ = 0.11, *p* = 0.74

The discriminant analysis returned univariate test results

**Table 4:** Percentage of trunks by tree species, trunk diameters, trunk densities, stem volumes, stem basal areas, and estimated mean tree age (mean ± 95% confidence intervals) of occupied (*N* = 78) and unoccupied (*N* = 54) ant sampling plots of 50 m radius

	Mean ± CI		Univariate test
	Occupied	Unoccupied	
*Picea abies* %	74.3 ± 3.9	71.8 ± 5.6	*F*_1, 130_ = 0.54, *p* = 0.46
*Pinus sylvestris* %	12.4 ± 3.1	12.9 ± 4.0	*F*_1, 130_ = 0.04, *p* = 0.83
*Betula* sp. %	10.5 ± 2.4	9.2 ± 2.9	*F*_1, 130_ = 0.45, *p* = 0.50
**Other deciduous trees %**	**2.9 ± 1.3**	**6.0 ± 2.7**	***F***_**1, 130**_** = 5.27, *p* = 0.023**
Mean trunk diameter (cm)	25.3 ± 0.8	25.0 ± 1.3	*F*_1, 130_ = 0.12, *p* = 0.73
Mean trunk density (ha^−1^)	973.7 ± 77.3	948.1 ± 89.8	*F*_1, 130_ = 0.18, *p* = 0.66
Mean stem volume (m^3^ ha^−1^)	323.1 ± 24.6	298.7 ± 24.5	*F*_1, 130_ = 1.84, *p* = 0.18
Mean stem basal area (m^2^ ha^−1^)	46.6 ± 2.7	43.5 ± 2.6	*F*_1, 130_ = 2.53, *p* = 0.11
Mean tree age (years)	49.3 ± 2.1	48.7 ± 3.2	*F*_1, 130_ = 0.12, *p* = 0.73

The occupied plots were occupied by either of *Formica aquilonia* or *F. polyctena*. The discriminant analysis returned univariate test results. Statistically significant difference is bolded

### Landscape habitat composition around colonies

**Fig. 2:**
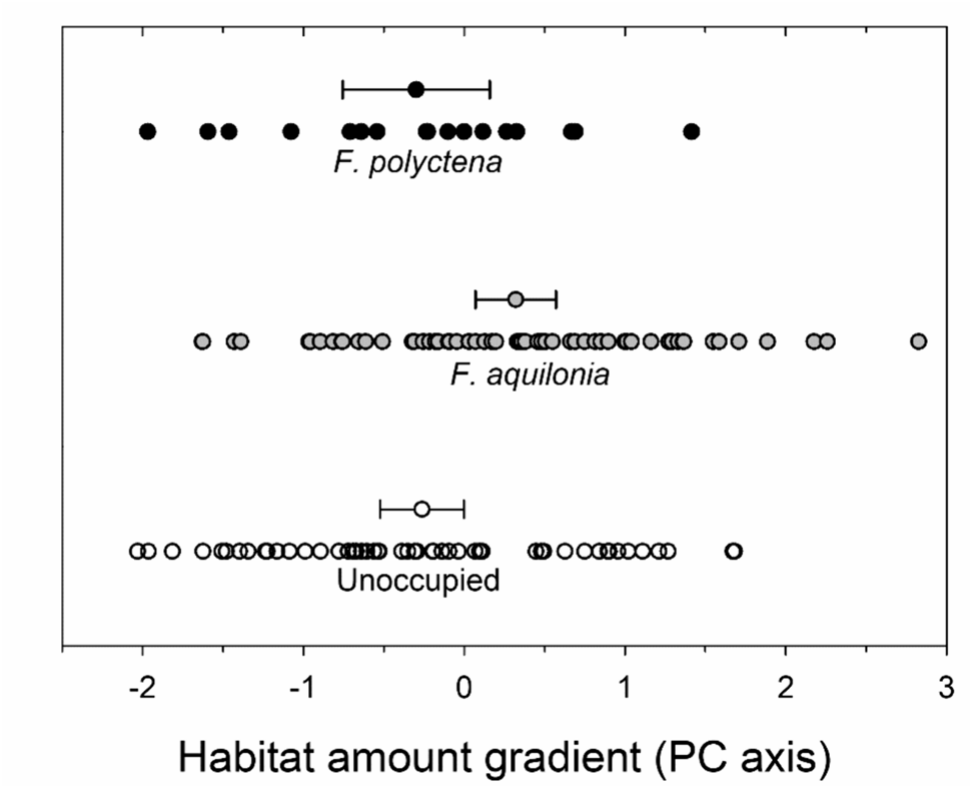
The distribution of the focal patches of *F. aquilonia* and *F. polyctena*, as well as the unoccupied forest patches along the gradient of forest habitat amount as indicated by the PC axis. A positive PC value indicates an increase in forest habitat percentage, old forest percentage, and focal patch size (ha). The habitat amount index at a radius of 500 m has a principal component loading (PC1)

In the landscape analyses of the amount of forest habitat (radius 500 m) around unoccupied and *F. aquilonia* and *F. polyctena* occupied sampling plots, there were significant differences (*F*_2, 130_ = 6.26, *p* = 0.0025; Fig. 2). *Formica aquilonia* colonies had a greater amount of continuous forest cover in their surroundings compared to unoccupied sites. The amount of continuous forest cover surrounding *F. polyctena* colonies did not differ from that of unoccupied sampling plots (Tukey’s multiple comparison adjustments, *F. aquilonia* vs. *F. polyctena*: *p* = 0.038; *F. aquilonia* vs. unoccupied: *p* = 0.0045; *F. polyctena* vs. unoccupied: *p* = 0.99; see Fig. 2).

The sizes of the focal patches differed significantly between patches with unoccupied, *F. aquilonia* occupied and *F. polyctena* occupied ant sampling plots (*F*_2, 130_ = 4.36, *p* = 0.015). The patches with *F. aquilonia* were the largest (38.3 ± 4.3 ha), followed by the unoccupied patches (30.1 ± 4.8 ha) and the patches occupied by *F. polyctena* (28.4 ± 7.3 ha) (Tukey’s tests: *F. aquilonia* vs. unoccupied: *p* = 0.034; *F. aquilonia* vs. *F. polyctena*: *p* = 0.059). There was no significant difference between the unoccupied and the *F. polyctena* occupied focal patches (*F. polyctena* vs. unoccupied: *p* = 0.92).

Since the landscape variables are not independent from each other’s the differences in landscape structure around the occupied sites were consequently analysed using single-predictor models. The ant sampling plots occupied by *F. aquilonia* located in larger focal patches, surrounded by a significantly higher proportion of forest land and a slightly higher proportion of ATF cover compared to patches occupied by *F. polyctena* (see Table 5). Further, the percentage of spruce forest cover was significantly greater in patches that were occupied by *F. aquilonia*. The cover of pine, deciduous and mixed forests, however, did not differ. Furthermore, the sites occupied by *F. polyctena* situated in more fragmented landscapes, i.e., higher mean neighbour patch distance, than *F. aquilonia* sites (Table 5).

**Table 5:** Estimated marginal means (±95% confidence limits) of landscape variables measured in 500 m of radius surroundings of focal patches occupied by *F. aquilonia* and *F. polyctena* along with the results of the generalized linear models (GLM)

	*F. aquilonia*	*F. polyctena*	GLM
% Forest cover	**77.4 (75.0–79.7)**	**71.7 (66.5–76.5)**	***F***_**1,76**_** = 4.50, *p* = 0.0372**
% ATF cover	**80.1 (78.3–81.8)**	**76.1 (72.4–79.7)**	***F***_**1,76**_** = 3.93, *p* = 0.0511**
Focal patch area (ha)	**38.3 (34.0–42.5)**	**28.4 (20.4–36.4)**	***F***_**1,76**_** = 4.68, *p* = 0.0336**
Mean nearest neighbour (m)	**32.8 (29.7–35.8)**	**42.1 (36.3–47.9)**	***F***_**1,76**_** = 8.11, *p* = 0.0057**
% Spruce forest cover	**55.9 (52.5–59.2)**	**47.4 (40.5–54.0)**	***F***_**1,76**_** = 5.19, *p* = 0.0255**
% Pine forest cover	37.4 (34.4–40.3)	36.7 (31.0–42.2)	*F*_1,76_ = 0.05, *p* = 0.8164
% Deciduous forest cover	14.8 (12.9–16.7)	17.0 (13.5–20.6)	*F*_1,76_ = 1.23, *p* = 0.2705
% Mixed forest cover	41.8 (39.8–43.8)	41.6 (37.9–45.3)	*F*_1,76_ = 0.01, *p* = 0.9410

ATF refers to at least advanced thinning forest with the total timber volume over 102 m^3^ ha^−1^. Forest cover refers to to coverage of forest with timber volume ≥50 m^3^ ha^−1^. Statistically significant differences are bolded

### Habitat amount-associated occupancy

**Fig. 3:**
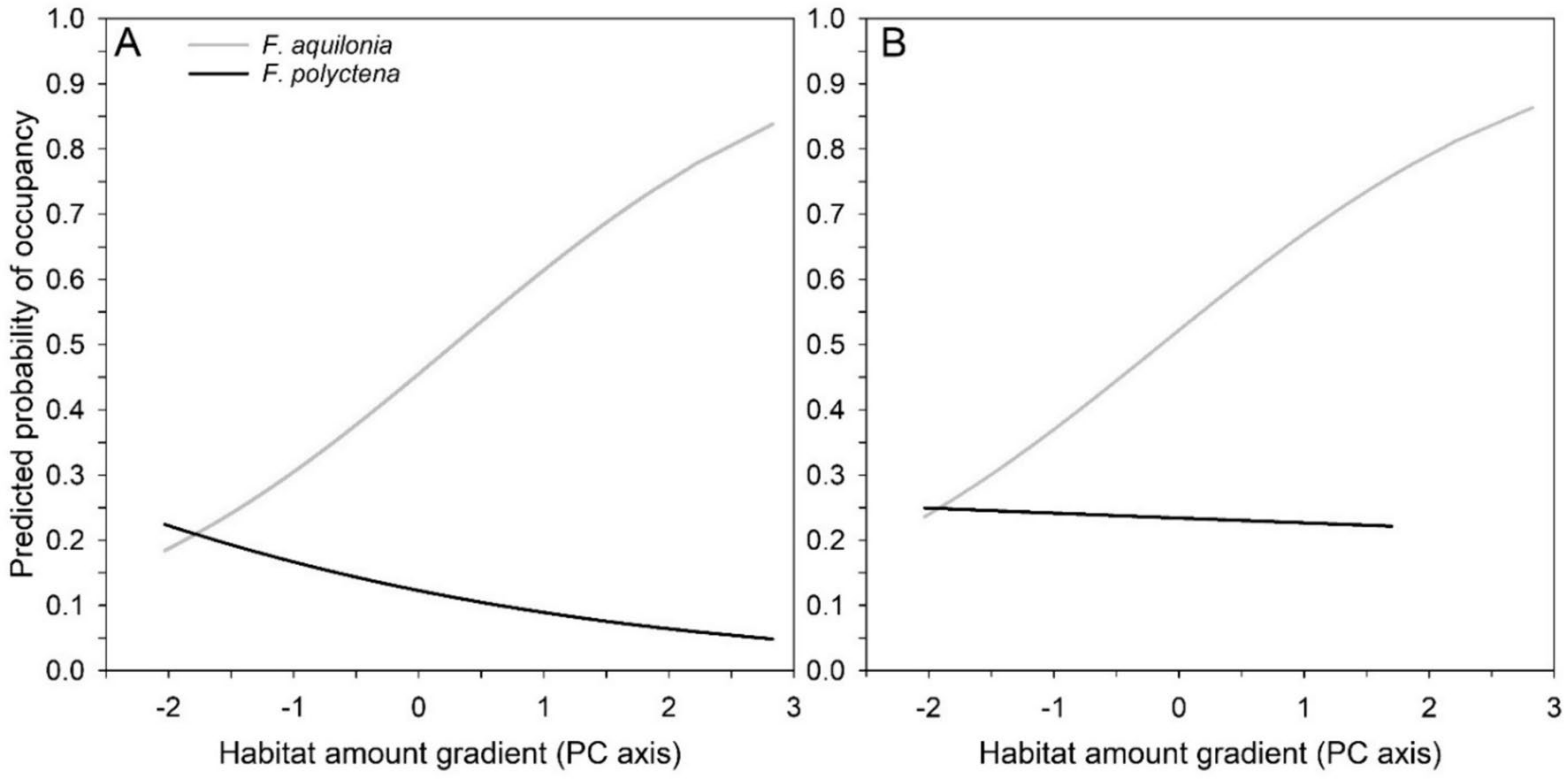
The predicted probability of occupancy for *F. aquilonia* and *F. polyctena* along the forest habitat amount gradient. Model predictions **A** are generated from the full dataset (competitor present model), while model predictions **B** are from data where competitor-occupied focal patches are excluded from the model (competitor-free model)

The occupancy rate of *F. aquilonia* in the sampling plots substantially increased with increasing habitat amount gradient. Conversely, the habitat amount gradient was not significantly associated with the occupancy rate of *F. polyctena* in the sampling plots (*F. aquilonia*: *F*_1, 131_ = 10.76, *p* = 0.0013; *F. polyctena*: *F*_1, 131_ = 1.73, *p* = 0.19; Fig. 3A). We conducted additional testing on the relationship between the species pair and the gradient of forest habitat amount excluding from the analysis sites that were occupied by the competitor species. The observed associations were generally like those in models including competitor-occupied patches; the occupancy rate of *F. aquilonia* in sampling plots increased with increasing habitat amount while no significant association was observed for *F. polyctena* and habitat amount (*F. aquilonia*: *F*_1, 114_ = 9.02, *p* = 0.0033; *F. polyctena*: *F*_1, 70_ = 0.02, *p* = 0.89; Fig. 3B). It is worth noting that the ant sampling plots in four largest forest patches were occupied by *F. aquilonia* and were therefore not available for *F. polyctena* in this data set (Figs. 2 and 3B).

## Discussion

The species pair *F. aquilonia* and *F. polyctena* should compete for food and nesting habitat resources, which was supported by our finding that although the ant species prefer similar forest characteristics, they never coexisted in a close distance. In our ant sampling plots *F. aquilonia* was almost four times more common than the competitor *F. polyctena* (61 vs. 17 occupied sampling plots, 146 nests vs. 20 nests). Although there were 55 unoccupied ant sampling plots, there should still have been sites where the species co-occur. Although forest characteristics were similar at a smaller spatial scale (50 m radius), at the landscape scale (500 m radius), sites occupied by *F. aquilonia* showed association with a greater amount of forest habitat and a higher amount of spruce dominated ATF and older forest patches in the surrounding matrix of a focal patch compared to *F. polyctena*. Unlike *F. aquilonia*, the occurrence of *F. polyctena* was not correlated with the amount of forest habitat. Our findings indicate that *F. polyctena* demonstrates greater tolerance to smaller forest fragments and longer nearest neighbour patch distances, while *F. aquilonia* displays a higher demand for more continuous forest landscape.

The established ecological theory (Volterra 1926; Lotka 1932) suggests that ecologically identical species cannot stably coexist. However, the extinction-recolonisation process is known to promote “fugitive coexistence” in patchy environments. It allows an inferior competitor that is a better coloniser to persist by exploiting the period between its patch colonisation and arrival of a superior competitor (Hanski 1983; Ruokolainen and Hanski 2016; Kawecki 2017). Theoretical models suggest that in systems with coexisting species, the equilibrium level and the proportion of patches that are doubly occupied are determined by the strength of competition on colonization and exclusion. Two asymmetrically competing species can coexist when there is a trade-off between local competition ability and invasion ability (Wang et al. 2000).

Wood ants within the *Formica rufa* group exhibit differences in their capacity to colonise and disperse, predominantly arising from varying colony life strategies: monogyny/monodomy versus polygyny/polydomy. The monogynous species demonstrates superior colonisation and dispersal flight abilities over a greater distance compared to polygynous species (Rosengren et al. 1993). There are no published data available on differences in the dispersal or colonization capacity of either study species, both of which are similarly polygynous and polydomous and territorial dominants.

Several theories propose that competitors may coexist in patchy habitats if there is a competitively superior species that poorly disperses and/or if the inferior species has a lower extinction rate (Nee and May 1992; Tilman 1994; Tilman et al. 1994; Moilanen and Hanski 1995). This equilibrium could persist if species inhabit different habitat patches that are separated by unsuitable areas, and gene flow occurs (Hanski 1983; Moilanen and Hanski 1995). The terms ‘superior’ and ‘inferior’ when describing competition in our ecologically similar species may not be precise. However, as *F. aquilonia* seems to be numerically dominant in continuous habitats, it may be considered the ‘superior competitor’ in this landscape and latitude. The ‘inferior’ *F. polyctena* may inhabit areas that *F. aquilonia* has left temporarily or permanently uninhabited, such as relatively small forest patches.

The numerical dominance by *F. aquilonia* can be due to several reasons that are mutually not exclusive: Firstly, *F. polyctena* as a southern species is less common just because it is at its northern limit. Secondly, *F. aquilonia* is the firstcomer and *F. polyctena* is now slowly colonising habitat patches that are unoccupied by *F. aquilonia*. Thirdly, *F. aquilonia* that has higher nest numbers (see Table 2) per occupied sampling sites has just lower habitat-level extinction rate compared to *F. polyctena*. Fourthly, *F. aquilonia* is behaviourally superior competitor compared to *F. polyctena*.

The first two options are supported by the fact that there are many empty patches available and that *F. polyctena* is a southern species and the third option is supported by our data. However, based on field observations *F. aquilonia* as a behaviourally superior competitor is doubtful. Colonies of *F. polyctena* tend to be highly populated, with wider active foraging trails around nest mounds in comparison to *F. aquilonia* (J.Sorvari unpublished). Additionally, the volume and diameter of nest mounds of *F. polyctena* tend to be larger than those of *F. aquilonia* (Wuorenrinne 1994; Punttila and Kilpeläinen 2009). Similarly, in our data, the mean basal area of nest mounds was also found to be larger in *F. polyctena* (Table 2). As both species appear to be equal competitors, the dominance of *F. aquilonia* in the studied landscape may be attributed to a lower rate of extinction caused by a higher degree of polydomy and/or improved adaptation to the northern latitudes.

Our result supports the comprehension that two equal competing species can co-exist in a patchy landscape if they share the environment differently in relation to e.g., the amount and fragmentation of a habitat (Hanski 1983). While the amount of forested habitat was higher in surrounding *F. aquilonia* sites compared to surrounding unoccupied and *F. polyctena* sites, the forest habitat amounts surrounding *F. polyctena* colonies did not differ from that of unoccupied sites. In addition, *F. aquilonia* showed a significant association with large forest patches, whereas *F. polyctena* occupied and unoccupied patches did not differ in size and were free from this kind of habitat size association in our data. This may be due to better nutrient levels in surrounding forests and large patches and/or a competitive strategy between species. In large patches, *F. aquilonia* may have had enough space to develop into strong colonies capable of excluding all other territorial species. These may indicate that despite of the current numerical dominance of patches by *F. aquilonia*, the landscape characteristics can eventually favour *F. polyctena* over *F. aquilonia* already in the current level of forest fragmentation. Punttila et al. (1994) and Punttila (1996) found out that polygynous wood ant species (*F. aquilonia*) are being replaced by monogynous species, especially by *F. lugubris* in fragmenting Finnish boreal forests. The study areas of Punttila did not include *F. polyctena*, so the landscape associations of the two polygynous species remained unanswered. Our study is the first to show the difference in area sensitivity between the top dominant polygynous/polydomous wood ants. Our data suggest that increasing fragmentation would disadvantage the more area-sensitive *F. aquilonia.*

Habitat loss and fragmentation are important factors determining population dynamics and spatial distribution. The most common forest management strategy in Finland is sequential clear-cutting with the aim of forest regeneration, followed by even-aged forest management (Vaahtera et al. 2023). Clear-cutting has adverse impacts on red wood ants, reducing the amount of food resources available to ant colonies (Punttila et al. 1991; Sorvari and Hakkarainen 2009; Sorvari et al. 2011) and disrupting nest temperature regulation (Sorvari and Hakkarainen 2009; Sorvari et al. 2016). In addition, such disturbances result in reduced reproduction of new queens and males, as well as reduced local population sizes (Sorvari and Hakkarainen 2005; 2007a, b). These studies by Sorvari et al. were carried out on *F. aquilonia*, but due to the similar diet, habitat, nest mounds, and colony structure, the effects are likely to be the same for *F. polyctena*. However, according to our results, *F. polyctena* could survive better in small and more isolated forest fragments left in intensively managed areas compared to *F. aquilonia*, and thus *F*. *polyctena* could better withstand the stress of forest management.

For species living in ephemeral (patchy) habitats, landscapes are highly dynamic rather than static. It has been shown that metapopulation persistence and extinction are strongly influenced by the rate at which the landscape changes, in addition to the amount of habitat destroyed (Keymer et al. 2000). It appeared that under competitive pressure a fugitive species resilience to disturbance or resource limitations may be more important than its ease of dispersal. The fugitive species experienced reduced competitive pressure in regions with reduced resources (i.e., marginal habitat), especially when its dispersal range is long relative to the superior competitor (Graniero 2007). Habitat destruction or patch removal, whether it is permanent or temporary, reduces the number (and proportion) of patches occupied by the superior competitor but can result in an increase in the total number of patches occupied by the inferior competitor (Hanski and Ranta 1983; Nee and May 1992; Moilanen and Hanski 1995).

Besides of forest fragmentation, climate change may favour the southern vicariant *F. polyctena* over its more northern counterpart, *F. aquilonia*. At present, *F. polyctena* is numerically dominant solely in a coastal region of southwestern Finland (Härkönen and Sorvari 2017; Sorvari 2021). South-western Finland belongs to the hemiboreal forest zone, while our study region is in the southern boreal zone. The zones differ in terms of temperature sums and forest characteristics. The hemiboreal zone serves as a transition zone between boreal and temperate zones and is characterised by a rising proportion of broadleaved trees such as the pedunculate oak (*Quercus robur*), which are mixed with otherwise boreal-type trees like conifers and birches. As a species belonging to the southern, temperate zone, *F. polyctena* appears to have superior adaptability to the resources available in hemiboreal forests, as opposed to boreal forest specialist *F. aquilonia*.

In course of time, the dominance of *F. polyctena* may expand into wider areas in southern Finland due to climate change induced changes in temperature regimes and alterations in tree species composition. Forest simulations have predicted a decline of Norway spruce in southern Finland with warming climate, as demonstrated by several studies (e.g., Ge et al. 2013; Torssonen et al. 2015; Ikonen et al. 2017, 2020). In the present data, *F. polyctena* is associated with lower proportion of Norway spruce compared to *F. aquilonia* and therefore may be better able to tolerate the decline of Norway spruce. Due to the impact of anthropogenic land use, forestry, and a warming climate, it appears that *F. polyctena* is being favoured over *F. aquilonia*. Consequently, we predict that *F. polyctena* will occupy a greater distribution in northern Europe, while the current dominance of *F. aquilonia* may decrease due to mounting competition with *F. polyctena* in warmer climates and more fragmented forests.

Hybridisation between species of *Formica rufa* group wood ants is prevalent in Finland, as supported by Pamilo et al. (1979), Sorvari (2006), Kulmuni et al. (2010), and Satokangas et al. (2023). Partial overlap during mating periods between *F. aquilonia* and *F. polyctena* could potentially lead to hybridisation events among these closely related species (Sorvari 2006). Similarly, overlapping mating periods are suggested to enable hybridisation between another red wood ant species pair, *F. polyctena* and *F. rufa* (Seifert 1991). Moreover, forest fragmentation appears to facilitate hybridization between different wood ant species (Seifert et al. 2010). Interestingly, the habitat patches hosting *F. polyctena* in our study were generally, but not restricted to smaller in size than those of *F. aquilonia*. Workers of *F. polyctena* and suspected hybrids between *F. polyctena* and *F. aquilonia* may exhibit intermediate phenotypic characteristics, as described by Sorvari (2006). However, at one specific site referred to as *F. polyctena*, these characteristics were visible, while all other sites showed typical phenotypes for both species, as observed by JS. Genomic evidence of hybridization events is common among wood ant species, but they can generally be classified into phenotypes of specific species using morphological markers (Satokangas et al. 2023). In Finland, *F. polyctena*-like hybrids typically inherit mitochondria from *F. polyctena* or the *F. polyctena*/*rufa* clade, as stated by Satokangas et al. (2023). Consequently, workers of the *F. polyctena* and those similar to *F. polyctena* (putative hybrids) were considered as *F. polyctena* in this study. In this species pairing, it has been proposed that hybridisation could prove advantageous for *F. polyctena*, which is situated at its northern range limit (Satokangas et al. 2023).

In conclusion, we first confirmed that the two species of large-scale wood ants, can competitively exclude each other and therefore cannot live near each other, suggesting the existence of strong interspecific competition. However, based on the observations by Kilpeläinen et al. (2008) and J. Sorvari, the two competing species may coexist in the same patch when the patch size is large enough to support spatially well separated (e.g., >100 m) multi-nest colonies of the competing species. Second, despite the potential for competitive exclusion, it is possible for these species to coexist in a fragmented forest landscape with variable habitat patch sizes, especially if the patch size distribution remains similar over time. However, the more widespread *F. aquilonia* is sensitive to forest cover and may lose landscape-level dominance to its more tolerant southern competitor, *F. polyctena*. This could be the result of increasing permanent or temporary loss of forest habitat, fragmentation, and the effects of climate change.

## Data Availability

Data used in the study are available from the corresponding author upon reasonable request.
